# Hemophagocytic lymphohistiocytosis: Unraveling the role of SARS-CoV-2 infection

**DOI:** 10.1016/j.gendis.2025.101764

**Published:** 2025-07-09

**Authors:** Erin Murphy, Krzysztof Data, Dominika Domagała, Julia Niebora, Artur Bryja, Małgorzata Józkowiak, Hanna Piotrowska-Kempisty, Piotr Dziegiel, Bartosz Kempisty, Paul Mozdziak

**Affiliations:** Graduate Physiology Program, North Carolina State University Campus, Box 7608, NC State, Raleigh, NC 27695, USA; Division of Anatomy, Department of Human Morphology and Embryology, Faculty of Medicine, Wroclaw Medical University, Wroclaw 50-368, Poland; Division of Anatomy, Department of Human Morphology and Embryology, Faculty of Medicine, Wroclaw Medical University, Wroclaw 50-368, Poland; Department of Toxicology, Poznan University of Medical Sciences, Poznań 61-701, Poland; Department of Toxicology, Poznan University of Medical Sciences, Poznań 61-701, Poland; Department of Basic and Preclinical Science, Institute of Veterinary Medicine, Nicolaus Copernicus University in Torun, Torun 87-100, Poland; Division of Histology and Embryology, Department of Human Morphology and Embryology, Faculty of Medicine, Wroclaw Medical University, Wroclaw 50-368, Poland; Division of Anatomy, Department of Human Morphology and Embryology, Faculty of Medicine, Wroclaw Medical University, Wroclaw 50-368, Poland; Physiology Graduate Faculty, North Carolina State University, Raleigh, NC 27607, USA; Institute of Veterinary Medicine, Department of Veterinary Surgery, Nicolaus Copernicus University, Torun 87-100, Poland; Center of Assisted Reproduction, Department of Obstetrics and Gynecology, University Hospital and Masaryk University, Brno 601 77, Czech Republic; Physiology Graduate Faculty, North Carolina State University, Raleigh, NC 27607, USA; Prestage Department of Poultry Science, North Carolina State University, Raleigh, NC 27607, USA

Hemophagocytic lymphohistiocytosis (HLH) is a rare immune disorder characterized by uncontrolled activation of cytotoxic T lymphocytes (T cells), natural killer cells (NK cells), triggering a cytokine burst, and severe systemic hyperinflammation.^1^ HLH can be inherited or developed from infection, autoimmune/autoinflammatory disorders, and neoplasms. The only effective treatment for familial HLH is hematopoietic stem cell transplantation. However, extensive research explores other cellular and acellular therapies. Acquired HLH can be treated by addressing its cause. The treatment of HLH includes chemotherapy, immunotherapy, steroids, antibiotics, and antiviral medications.

The diagnosis of HLH varies case by case, relying on symptoms, physical examination, and laboratory testing. Physical manifestations of the disease include prolonged fever, enlarged spleen or liver, rash, irritability, and seizures.

HLH is diagnosed based on the HLH-2004 criteria, requiring at least five of eight parameters: 1) fever ≥ 38.5 °C, 2) splenomegaly, 3) cytopenia, with at least two of the following: hemoglobin < 9 g/dL; platelets < 100,000/μL; absolute neutrophil count (ANC) < 1000/μL, 4) hypertriglyceridemia (>265 mg/dL) and/or hypofibrinogenemia (<150 mg/dL), 5) hemophagocytosis in bone marrow, spleen, lymph node, or liver, 6) low/absent NK cell activity, 7) ferritin > 500 ng/mL, 8) elevated soluble CD25 (soluble IL-2 receptor alpha) two standard deviations above age-adjusted laboratory-specific norms.

In the diagnosis, there is a division between the origin of the pathogenesis, into primary and secondary HLH. Primary HLH is driven by genetic inheritance, mutations, related signaling pathways, and innate immune cell damage. The familial form of HLH is usually inherited in an autosomal recessive manner and results from mutations in genes that regulate immune system function, such as *PRF1*, *UNC13D*, *STX11*, and *STXBP2*, affecting cytotoxic cell function, granule release, and vesicle trafficking in immune responses. Secondary HLH arises from diseases, malignancies, side effects of immunological therapies, and other external triggers. Crucial for pathophysiology are viral infections, including SARS-CoV-2.

The acute phase or the peak of symptoms of COVID-19 infection has been observed to trigger the onset of secondary HLH; with remission of HLH following the COVID-19 recovery period^2^ (Table S1). However, in the case of a 72-year-old female, recovery from HLH was temporary. After treatment with the steroid and chemotherapy combination, her condition improved significantly, only to decline severely within a 1.5-month period. She underwent a second round of HLH treatment, but it was not successful.^2^ This patient with well-controlled rheumatoid arthritis and a recent COVID-19 was admitted with worsening shortness of breath and diagnosed with acute on chronic systolic heart failure, kidney injury, anemia, and thrombocytopenia. The patient's condition rapidly deteriorated, resulting in fever, pancytopenia, acute liver injury, respiratory failure requiring mechanical ventilation, and renal failure. Laboratory findings, including hemoglobin of 7.9 g/dL, platelets of 50 Th/uL, ANC of 1.38 Th/μL, lactate dehydrogenase (LDH) of 6000 IU/L, ferritin > 10,000 ng/mL, fibrinogen of 136 mg/dL, and triglycerides of 656 mg/dL, suggested HLH, confirmed by an elevated soluble interleukin-2 receptor level of 57,026.9 pg/mL and a bone marrow biopsy showing hemophagocytosis. Treatment with the HLH-94 protocol (etoposide and dexamethasone) resulted in initial improvement, extubation, and liver function recovery, although she remained cytopenic and required transfusions. By week six, despite dialysis and improved inflammatory markers, her condition worsened, prompting treatment with ruxolitinib and dexamethasone. After developing an acute abdominal visceral perforation, she was placed on supportive care due to poor prognosis.^2^

In another case, a 65-year-old male was diagnosed with HLH following a prior COVID-19 infection, wherein he was intubated due to rapidly worsening respiratory condition^3^ (Table S1). He presented with a high fever (40.5 °C), dry cough, and hypoxemia, requiring oxygen support. Laboratory studies showed thrombocytopenia (platelets 81,000/μL), acute kidney injury (creatinine 1.28 mg/dL, blood urea nitrogen 33 mg/dL), mild transaminitis, lactic acidosis, and elevated C-reactive protein (CRP) (152 mg/L). His condition worsened, leading to thrombocytopenia, leukopenia, renal dysfunction, and respiratory failure requiring intubation. Shortly after the prolonged fever symptom from HLH began, the patient exhibited multiple organ dysfunction that escalated quickly. A computed tomography (CT) scan revealed bronchial wall thickening, pulmonary nodules, v enlarged periaortic lymph nodes, and hepatic steatosis. Following the worsening respiratory condition, the patient was transitioned to the intensive care unit (ICU), where a chest X-ray revealed bilateral infiltrates or substances that are thicker than air present in the lungs, which is associated with acute respiratory distress syndrome, or acute respiratory distress syndrome (ARDS). Further labs demonstrated highly elevated LDH (556 U/L), ferritin (25,570 ng/mL), hypertriglyceridemia (232 mg/dL), and worsening liver dysfunction. A bone marrow biopsy confirmed hemophagocytosis.

Researchers employed a line graph to track the key laboratory parameters, including white blood cell (WBC) count, LDH, fibrinogen, and triglyceride levels during hospitalization. The patient exhibited profound hyperferritinemia, with ferritin peaking at 98,261 ng/mL, and severe hypertriglyceridemia, with triglycerides rising to 1249 mg/dL. LDH reached 1282 U/L, while fibrinogen declined to 107 mg/dL. Transaminase levels were markedly raised, with AST peaking at 443 U/L and ALT at 1021 U/L, alongside conjugated hyperbilirubinemia reaching 16.7 mg/dL. The patient began the HLH-94 protocol with etoposide and dexamethasone, but complications including tumor lysis syndrome, acute tubular necrosis requiring dialysis, disseminated intravascular coagulation, and refractory thrombocytopenia limited treatment. The patient was transferred to a tertiary center for IL-2 receptor blocker therapy, but his prognosis remained poor, underscoring HLH severity in post-COVID-19 patients.^3^

Clinicians often struggle to distinguish HLH promptly due to its similarity to COVID-19 infection. The manifestation of COVID-19 symptoms varies case-by-case, while more severe clinical symptoms can be linked to later HLH diagnosis. Although reported cases are limited, persistent inflammation, immune dysfunction, or severe acute respiratory syndrome from COVID-19 may trigger secondary HLH. Another resemblance is between HLH and sepsis accompanied by fever and multiple organ dysfunction.^3^ However, the exact pathophysiology of post-COVID-19-induced HLH remains unclear. A proposed connection between COVID-19 and acquired HLH has been proposed by Fadlallah et al,^4^ as shown in Figure 1. The cytokine storm, or hypercytokinemia, is a severe immune response involving excessive cytokine release, a hallmark of HLH. While HLH is a specific disorder with defined genetic and immune dysfunction, a cytokine storm is a broader immunological response seen in HLH, severe infections, autoimmune diseases, and COVID-19. However, both involve excessive immune activation and an uncontrolled pro-inflammatory cytokine release, causing severe inflammation and tissue damage. In extreme COVID-19 cases, cytokine storm is observed and manifests as inflammation. The proposed pathophysiological pathway between COVID-19 and HLH is that the severe SARS-CoV-2 infection triggers the production and release of proinflammatory cytokines. The virus binds to the angiotensin-converting enzyme-2 (ACE2) receptor, blocking single-stranded ribonucleic acid (ssRNA). This ssRNA is essential for nuclear factor kappa light chain enhancer of activated B cells (NF-κB) in the pathway, leading to a cytokine storm.^5^ This causes a domino effect in the immune system by overproducing interleukin-1beta IL-1β, CD4^+^ T cells, activated NK cells, and other macrophages in the body. This process reflects the biphasic nature of COVID-19 and the inflammatory response, suggesting a molecular mimicry between self-antigen and SARS-CoV-2.

**Figure 1:**
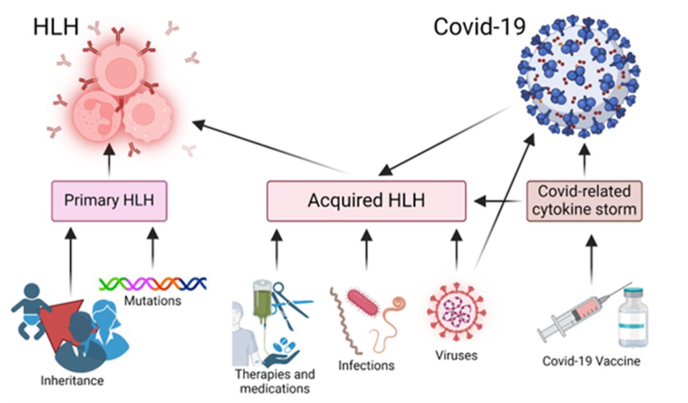
Pathogenetic relations between hemophagocytic lymphohistiocytosis (HLH) and COVID-19 diseases.

In summary, HLH is a rare inflammatory disorder affecting the immune system. Acquired HLH symptoms generally presented in adults often vary, making diagnosing HLH challenging. With timely diagnosis and treatment, it is possible to live beyond the 41% mortality rate of HLH. COVID-19 and HLH are linked through a cytokine storm causing severe inflammation, a hallmark of HLH.

## CRediT authorship contribution statement

**Erin Murphy:** Writing – original draft. **Krzysztof Data:** Writing – original draft. **Dominika Domagała:** Writing – original draft. **Julia Niebora:** Writing – original draft. **Artur Bryja:** Supervision, Writing – review & editing. **Małgorzata Józkowiak:** Visualization, Writing – original draft. **Hanna Piotrowska-Kempisty:** Supervision, Conceptualization. **Piotr Dziegiel:** Project administration, Supervision. **Bartosz Kempisty:** Conceptualization, Supervision, Project administration, Writing – review & editing. **Paul Mozdziak:** Project administration, Writing – review & editing, Funding acquisition.

## Funding

This research was partly funded by the U.S. Department of Agriculture's (USDA) Animal Health Project NC 07090.

## Conflict of interests

The authors have no conflicts of interest to declare that are relevant to the content of this article.
