# Supplementary material for: Hemophagocytic lymphohistiocytosis: Unraveling the role of SARS-CoV-2 infection
Source: Genes Dis. 2025 Jul 9;13(1):101764. doi: 10.1016/j.gendis.2025.101764 (PMC12447879; doi:10.1016/j.gendis.2025.101764)
Supplement: Multimedia component 1 [file mmc1.docx]

**Literature Search Strategy**

A systematic literature search was conducted using the PubMed database to identify relevant case reports describing hemophagocytic lymphohistiocytosis (HLH) following COVID-19 infection. The search included articles published from 2020 to 2024.

We used combinations of the following keywords and MeSH terms:

- “Hemophagocytic lymphohistiocytosis” OR “HLH”,
- “COVID-19” OR “SARS-CoV-2”,
- “Post-COVID” OR “Recovered COVID” OR “long COVID”,
- “Case report” OR “Clinical case”.

**Inclusion Criteria:**

- Case reports. HLH developed after recovery from COVID-19.
- Studies reporting clinical, laboratory, and treatment data sufficient for comparison.
- Adult patients (≥18 years).

**Exclusion Criteria:**

- Studies reporting HLH during the acute phase of COVID-19 infection.
- Articles focused on pediatric populations.
- Reviews, editorials, or commentaries without primary patient data.
- Cases where HLH was clearly attributed to other causes (e.g., malignancy, genetic HLH, autoimmune flare) independent of COVID-19.

**Table S1. Comparative Table of Two Post-COVID HLH Cases ^2,3^**

| **Parameter** | **Patient 1 ^2^** | **Patient 2 ^3^** |
| --- | --- | --- |
| **Age / Sex** | 72 / Female | 65 / Male |
| **COVID-19 history** | 2 months prior, no specific treatment mentioned | 1 month prior, mild case, treated with Paxlovid |
| **Comorbidities** | Well-controlled rheumatoid arthritis | Not reported |
| **Initial symptoms** | Progressive shortness of breath | Fever, dry cough, fatigue |
| **Initial diagnoses** | Acute on chronic heart failure, AKI, anemia, thrombocytopenia | Suspected pneumonia, AKI, mild transaminitis, lactic acidosis |
| **Day of HLH progression** | Day 9 of hospitalization | Within initial hospitalization |
| **Organ failure** | Multiorgan failure (respiratory, liver, renal) | Multiorgan failure, worsening thrombocytopenia, leukopenia, and renal dysfunction |
| **Bone marrow biopsy** | Hemophagocytosis, no malignancy | Hemophagocytosis, no malignancy |
| **PET/CT** | No lymphoma detected | No lymphoma detected |
| **HLH diagnosis criteria** | HLH-2004 criteria met | HLH-2004 criteria met |
| **Initial treatment** | HLH-94 protocol, dexamethasone + etoposide | Dexamethasone, planned etoposide and anti-IL-2R agent |
| **Response to 1st line therapy** | Initial clinical improvement (extubated, off dialysis) | Incomplete, ongoing disease activity |
| **Relapse timing** | 6–8 weeks after initial treatment | Not specified |
| **2nd line therapy** | Ruxolitinib 5 mg BID + dexamethasone 2 mg/day | Not reported (anti-IL-2R planned) |
| **Transplant eligibility** | Not suitable for stem cell transplant | Not mentioned |
| **Outcome** | Rehospitalized with respiratory failure and gut perforation; transitioned to palliative care | Ongoing inflammation, persistent disease |
